# Analysis of 4,664 high-quality sequence-finished poplar full-length cDNA clones and their utility for the discovery of genes responding to insect feeding

**DOI:** 10.1186/1471-2164-9-57

**Published:** 2008-01-29

**Authors:** Steven G Ralph, Hye Jung E Chun, Dawn Cooper, Robert Kirkpatrick, Natalia Kolosova, Lee Gunter, Gerald A Tuskan, Carl J Douglas, Robert A Holt, Steven JM Jones, Marco A Marra, Jörg Bohlmann

**Affiliations:** 1 Michael Smith Laboratories, University of British Columbia, Vancouver, British Columbia, V6T 1Z4, Canada; 2 British Columbia Cancer Agency Genome Sciences Centre, Vancouver, British Columbia, V5Z 4E6, Canada; 3 Department of Botany, University of British Columbia, Vancouver, British Columbia, V6T 1Z4, Canada; 4 Environmental Sciences Division, Oak Ridge National Laboratory, Oak Ridge, Tennessee, 37831, USA; 5 Department of Forest Sciences, University of British Columbia, Vancouver, British Columbia, V6T 1Z4, Canada; 6 Department of Biology, University of North Dakota, Grand Forks, North Dakota, 58202-9019, USA

## Abstract

**Background:**

The genus *Populus *includes poplars, aspens and cottonwoods, which will be collectively referred to as poplars hereafter unless otherwise specified. Poplars are the dominant tree species in many forest ecosystems in the Northern Hemisphere and are of substantial economic value in plantation forestry. Poplar has been established as a model system for genomics studies of growth, development, and adaptation of woody perennial plants including secondary xylem formation, dormancy, adaptation to local environments, and biotic interactions.

**Results:**

As part of the poplar genome sequencing project and the development of genomic resources for poplar, we have generated a full-length (FL)-cDNA collection using the biotinylated CAP trapper method. We constructed four FLcDNA libraries using RNA from xylem, phloem and cambium, and green shoot tips and leaves from the *P. trichocarpa *Nisqually-1 genotype, as well as insect-attacked leaves of the *P. trichocarpa *× *P. deltoides *hybrid. Following careful selection of candidate cDNA clones, we used a combined strategy of paired end reads and primer walking to generate a set of 4,664 high-accuracy, sequence-verified FLcDNAs, which clustered into 3,990 putative unique genes. Mapping FLcDNAs to the poplar genome sequence combined with BLAST comparisons to previously predicted protein coding sequences in the poplar genome identified 39 FLcDNAs that likely localize to gaps in the current genome sequence assembly. Another 173 FLcDNAs mapped to the genome sequence but were not included among the previously predicted genes in the poplar genome. Comparative sequence analysis against *Arabidopsis thaliana *and other species in the non-redundant database of GenBank revealed that 11.5% of the poplar FLcDNAs display no significant sequence similarity to other plant proteins. By mapping the poplar FLcDNAs against transcriptome data previously obtained with a 15.5 K cDNA microarray, we identified 153 FLcDNA clones for genes that were differentially expressed in poplar leaves attacked by forest tent caterpillars.

**Conclusion:**

This study has generated a high-quality FLcDNA resource for poplar and the third largest FLcDNA collection published to date for any plant species. We successfully used the FLcDNA sequences to reassess gene prediction in the poplar genome sequence, perform comparative sequence annotation, and identify differentially expressed transcripts associated with defense against insects. The FLcDNA sequences will be essential to the ongoing curation and annotation of the poplar genome, in particular for targeting gaps in the current genome assembly and further improvement of gene predictions. The physical FLcDNA clones will serve as useful reagents for functional genomics research in areas such as analysis of gene functions in defense against insects and perennial growth. Sequences from this study have been deposited in NCBI GenBank under the accession numbers EF144175 to EF148838.

## Background

Poplars are keystone tree species in several temperate forest ecosystems in the Northern Hemisphere. Poplars are also intensively cultivated in plantation forestry for the production of wood, pulp, and paper. Fast growing poplars can serve functions in phytoremediation, as a sink for carbon sequestration, and as a feedstock for biofuel production. Poplar has also been firmly established as a model research system for long-lived woody perennials (reviewed in [1]). Advances in functional genomics of poplar have been greatly enhanced by the availability of a high-quality genome sequence from *P. trichocarpa *(Nisqually-1; [2]), combined with comprehensive genetic [3-6] and physical genome [7] maps, as well as the availability of several platforms for transcriptome analysis [8-11] and genetic transformation. Large collections of expressed sequence tags (ESTs) have also been developed from a variety of poplar species and hybrids focussing on gene discovery in wood formation, dormancy, floral development and stress response [9,11-20]. These short, single-pass EST reads have been a critical resource for gene discovery, genome annotation, and the construction of microarray platforms.

High-accuracy, sequence-verified FLcDNA sequences that span the entire protein-coding region of a given gene can advance comparative, functional, and structural genome analysis. For example, the accuracy of *ab initio *prediction of protein-coding regions in genome sequences is limited by the difficulty of finding islands of coding sequences within an ocean of non-coding DNA, and by the complexity of individual genes that may code for multiple peptides through alternative splicing. More robust approaches that unambiguously identify protein-coding regions in a genome sequence have used FLcDNA data, as demonstrated for example in *Arabidopsis thaliana *[21-23]. Despite their immense value, sequence-verified FLcDNA clones, where multiple passes verify the authenticity of reads, have not been generated in most plant species subjected to genomic analysis. Only a few large FLcDNA data sets have been generated for plants; namely for rice [24], Arabidopsis [25], and maize [26,27]. In contrast, as of September 2007, there were only 1,409 complete sequences from individual poplar FLcDNA clones in the non-redundant (NR) division of GenBank, in addition to a larger number of putative full-length sequences assembled from EST reads of multiple cDNA clones.

Our poplar FLcDNA program in the areas of forest health genomics and wood formation has focused on mechanisms of defense and resistance against insects and genes associated with xylem development. The forest tent caterpillar (*Malacosoma disstria*; FTC; [28]) is a major insect pest that threatens the productivity of natural and plantation forests. Poplars deploy an array of combined defense strategies against herbivores that can be grouped as chemical and physical defenses, direct and indirect defenses, constitutive and induced defenses, as well as local and systemic defenses (reviewed in [29]). Several recent studies have been conducted on the molecular mechanisms underlying inducible defenses against herbivores in poplar [11,18,30-37].

In this paper, we report on the development of four FLcDNA libraries from poplar that served as the starting template for creating a substantial genomic resource of 4,664 sequence-verified FLcDNAs. We describe the overall structural features of these FLcDNA clones, annotation based on comparisons with other species, and the identification of 536 putative poplar-specific transcripts. Mapping the FLcDNA collection to the poplar genome sequence confirmed the overall high quality of the assembled genome sequence as well as the high quality of the FLcDNA resource, while also identifying 39 expressed poplar transcripts that appear to be derived from gap regions of the current genome sequence assembly and 173 new poplar genes that have not previously been identified in the genome assembly. By mapping 3,854 FLcDNAs to a poplar 15.5 K cDNA microarray platform and performing a comparison with existing transcriptome data, we identified 153 FLcDNAs that match transcripts differentially expressed following insect attack by FTC on poplar leaves.

## Results

### Selection and sequence finishing of FLcDNAs

FLcDNAs are defined as individual cDNA clones that contain the complete protein-coding sequence and at least partial 5' and 3' untranslated regions (UTRs) for a given transcript. This definition distinguishes *bona fide *FLcDNAs from *in silico *assembled EST sequences derived from multiple cDNA clones. In the latter case, it is possible that multiple, closely related genes or allelic variants of the same gene are assembled into a single consensus sequence. This problem is avoided when only sequences derived from the same physical FLcDNA clone are assembled. We prepared four FLcDNA libraries using the biotinylated CAP trapper method [38]. Three libraries constructed from xylem, phloem and cambium, and green shoot tips and leaves were derived from the *P. trichocarpa *Nisqually-1 genotype, for which the genome sequence has been reported [2]. An additional library was developed from the *P. trichocarpa *× *P. deltoides *hybrid H11–11 genotype using leaves subjected to FTC herbivory (Table 1).

**Table 1:** Libraries, tissue sources and species for sequences described in this study

cDNA Library	Tissue/Developmental Stage	Species (genotype)
PT-X-FL-A-1	Outer xylem^a^.	*Populus trichocarpa *(Nisqually-1)
PT-P-FL-A-2	Phloem and cambium^a^.	*P. trichocarpa *(Nisqually-1)
PT-GT-FL-A-3	Young and mature leaves, along with green shoot tips^a^.	*P. trichocarpa *(Nisqually-1)
PTxD-IL-FL-A-4	Local and systemic (above region of feeding) mature leaves harvested after continuous feeding by forest tent caterpillars, *Malacosoma disstria*. Local tissue was collected 4, 8 and 24 h post-treatment and systemic tissue 4, 12 and 48 h post-treatment^b^.	*P. trichocarpa *× *deltoides *(H11–11)

^a^Harvested May 15th, 2001 from eight year old trees within the Boise Cascade region of Washington state.

^b^One or two year old saplings grown in potted soil under greenhouse conditions at the University of British Columbia.

**Figure 1:**
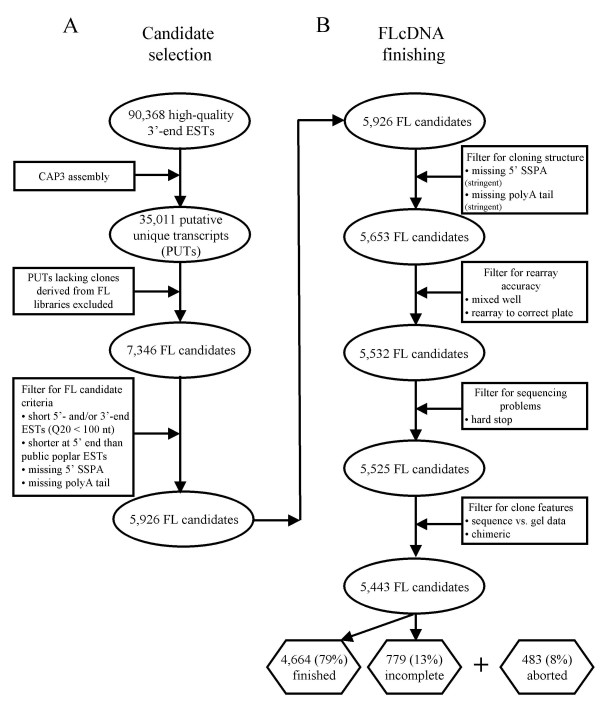
**Schematic of clone selection and complete insert sequencing of 4,664 FLcDNAs**. CAP3 assembly of 90,368 high-quality 3'-end ESTs identified 35,011 putative unique transcripts (PUTs) for the identification of candidate FLcDNAs. Only those PUTs containing at least one clone from a FLcDNA library were considered further. To maximize the number of FLcDNAs captured, candidate clones were excluded from further analysis if: (1) the 5' second strand primer adaptor (SSPA) was absent; (2) a polyA tail was absent; (3) 5'- and/or 3'-end ESTs had a Phred20 quality length (Q20) of < 100 nt; or (4) BLASTN (E < 1e^-80^) versus poplar ESTs in the public domain identified a candidate as potentially truncated (i.e., > 100 nt shorter) at the 5' end of the transcript relative to a matching EST. Among the 5,926 candidates selected for sequencing, only 483 (8%) were aborted at various stages of the sequence finishing pipeline due to: (1) missing cloning structures; (2) errors in re-array of glycerol stocks; (3) problematic sequencing such as hard stops; or (4) problematic clone features such as chimeric sequences. Through a combination of end reads and gap closing using primer walking, 4,664 (79%) sequence-verified FLcDNAs were completed. An additional 779 clones (13%) from the starting set of 5,926 will be finished in future work.

**Figure 2:**
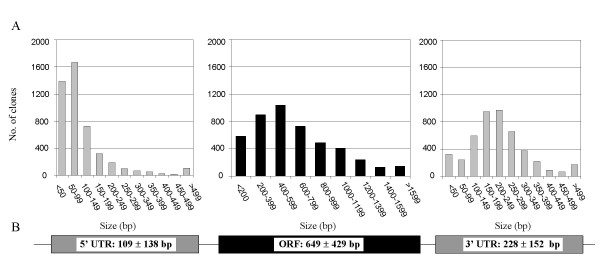
**Distribution of open reading frame (ORF) and 5' and 3' untranslated region (UTR) sizes among the finished 4,664 FLcDNAs (A), and the mean ORF and UTR length (± standard deviation) (B)**. Each finished FLcDNA sequence was examined for the presence of ORFs using either the EMBOSS getorf program (version 2.5.0; [55]) or an in-house BLAST-aided program. The getorf program identifies the longest stretch of uninterrupted sequence between a start (ATG) and stop codon (TGA, TAG, TAA) in the 5' to 3' direction for the predicted ORF. The BLAST-aided program detects ORFs by finding the starting methionine and stop codon in a poplar FLcDNA sequence relative to the same features in the most closely related Arabidopsis protein identified by BLASTX (E values < 1e^-20^). For this study, ORFs identified by the BLAST-aided method were utilized except in cases where the FLcDNA sequence did not show high similarity to an Arabidopsis protein, in which case the ORF identified by the getorf program was chosen. The presence and coordinates of the 5' second strand primer adaptor sequence (SSPA) and polyA tail were also noted. The regions between the 5'SSPA and the predicted ORF start and between the predicted ORF stop and the polyA tail were taken to be the 5' and 3' UTRs, respectively. The 5' SSPA and 3' polyA tail lengths were not included when determining UTR length.

To select candidate FLcDNAs for complete insert sequencing, we used a previously described bioinformatic pipeline for EST processing [11]. An initial set of 26,112 3' ESTs derived from FLcDNA libraries was combined with 81,407 3' ESTs from standard EST libraries [11] to generate a starting set of 107,519 3'-end ESTs, which resulted in 90,368 high-quality ESTs after filtering to remove sequences of low quality and contaminant sequences from yeast, bacteria and fungi. These sequences were then clustered using the CAP3 assembly program ([39]; assembly criteria: 95% identity, 40 bp window) to identify a set of 35,011 putative unique transcripts (PUTs; Figure 1). To maximize the capture of complete open reading frames (ORFs) and UTRs, only clones from full-length libraries were considered further. Using this strategy, we identified 5,926 cDNA candidate clones for full insert sequencing, which resulted in 4,664 sequence-verified poplar FLcDNA clones (see Additional file 1 and Figure 2). Inserts of 2,672 clones were completely sequenced using end reads only, with an average sequenced insert size of 735 ± 434 bp (average ± SD) and required an average of 4.5 ± 1.3 end reads to finish to high sequence quality. Using a combination of end reads and primer walking, inserts of an additional 1,992 clones were completely sequenced, with an average insert size of 1,308 ± 567 bp requiring 5.9 ± 2.8 end reads and 3.4 ± 1.8 internal primer reads per clone.

Analysis of the 4,664 FLcDNA sequences using the CAP3 clustering and assembly program ([39]; assembly criteria: 95% identity, 40 bp window) identified 3,505 FLcDNAs as unique singletons, with the remaining 1,159 grouping into 485 contigs, suggesting a total of 3,990 unique genes represented with finished FLcDNA sequences. The high percentage of unique transcripts (85.5%) within this set confirms the successful clone selection strategy (Figure 1) for establishing a low-redundancy clone set prior to sequence finishing.

### Sequence quality and "full-length" assessment of poplar FLcDNAs

**Figure 3:**
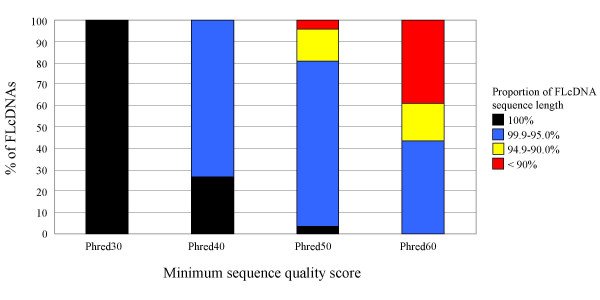
**Validation of sequence quality of FLcDNAs**. Sequence accuracy was measured as the percentage of the 4,664 FLcDNAs which, with 100%, 95.0–99.9%, 90.0–94.9% or < 90.0% of their sequence length, exceeded Phred30, Phred40, Phred50 or Phred60 sequence quality thresholds. All 4,664 FLcDNAs exceeded the Phred30 quality thresholds (calculated as less than 1 error in 10^3 ^sequenced nucleotides) over 100% of their sequence length. Even at the threshold level of Phred60 (calculated as less than 1 error in 10^6 ^sequenced nucleotides) the majority (61.2%) of the FLcDNA sequences met this very high sequence quality score over > 90.0% of their length.

All 4,664 finished FLcDNAs achieved a minimum of Phred30 (i.e., one error in 10^3 ^bases) sequence quality at every base. The majority of FLcDNAs were of even higher quality with the minimum and average Phred values exceeding Phred45 (i.e., one error in 3 × 10^4 ^bases) and Phred80 (i.e., one error in 10^8 ^bases), respectively (Figure 3). We predicted the complete protein-coding ORFs for all 4,664 FLcDNAs. The distribution of 5' UTR, ORF and 3' UTR lengths is illustrated in Figure 2 [also see Additional file 1]. The average sequenced FLcDNA length (from the beginning of the 5' UTR to the end of the polyA tail) was 1,045 ± 475 bp (mean ± SD), and ranged from 147 to 3,342 bp, whereas the average predicted ORF was 649 ± 429 bp and ranged from 33 to 2,935 bp. ORFs could not be detected (i.e., 30 bp or less) for 96 FLcDNAs. The 5' and 3' UTRs averaged 109 ± 138 bp and 228 ± 152 bp, respectively. These results are comparable to CAP trapper FLcDNA collections from other plant species including maize (cDNA insert 799 bp, 5' UTR 99 bp, 3' UTR 206 bp; [27]), Arabidopsis (cDNA insert *ca*. 1.2 kb; [40]) and rice (5' UTR 259 bp, 3' UTR 398 bp; [24]). Similarly, the average transcript length of the 45,555 poplar reference genes predicted *ab initio *from the genome sequence was 1,079 bp and 5' and 3' UTRs averaged 92 bp [2], in close agreement with our results obtained with FLcDNAs.

**Figure 4:**
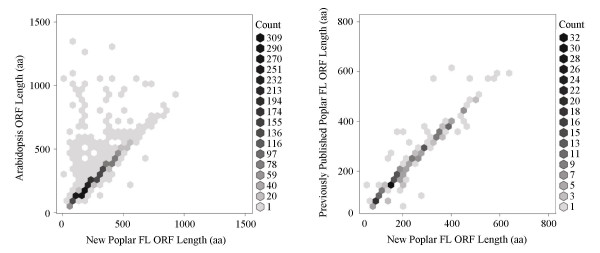
**Validation of poplar FLcDNAs by comparison to reciprocal BLAST matches against Arabidopsis peptides and previously published poplar FLcDNAs**. The set of 4,664 poplar FLcDNAs were compared using BLASTX to both The Arabidopsis Information Resource (TAIR) non-redundant Arabidopsis peptide set (28,952 sequences [56]) and a collection of 1,409 previously published poplar sequences from the non-redundant (NR) division of GenBank ([57], the NR release of December 19^th^, 2006) annotated as full-length (excluding predicted proteins derived from genomic DNA). FLcDNAs were excluded from the analysis when the in-house BLAST-aided ORF detection software identified a FLcDNA as problematic according to the following categories: truncation at the 5'-end (319), truncation at the 3'-end (50), frameshift (12), stop codon in the middle of an ORF (9), or inverted insert (3) [see Additional file 1]. No problematic features were identified in the remaining 4,271 FLcDNAs. This comparison identified 2,774 homologous Arabidopsis-poplar pairs and 288 homologous poplar transcript pairs. A FLcDNA pair was considered homologous if (1) the top BLASTX match exceeded a stringent threshold (% identity ≥ 50%; expect value ≤ 1e^-20^) and (2) the reciprocal TBLASTN analysis identified the same poplar FLcDNA with a score value equal to or within 10% of the top match. ORF lengths for Arabidopsis and public poplar sequences were extracted from the TAIR and NR records, respectively, and poplar ORF lengths from this study were predicted using either the EMBOSS getorf or in-house BLAST-aided programs (see Figure 2 legend). The greyscale shading of each hexagon represents poplar FLcDNA abundance. ORF lengths for three Arabidopsis-poplar pairs and eight homologous poplar transcript pairs differed by more than 500 aa and are not included in the figure.

To further assess the quality of the 4,664 poplar FLcDNAs, we performed reciprocal BLAST analysis against peptide sequences in The Arabidopsis Information Resource (TAIR) and against a set of 1,409 poplar sequences previously identified to be full-length (collected from the NR division of GenBank). Reciprocal BLAST analysis was performed with a stringent similarity threshold [% identity ≥ 50%; expect (E) value ≤ 1e^-20^] and identified 2,774 and 288 pairs, respectively, with Arabidopsis and previously published poplar FLcDNAs (Figure 4). Of the 288 homologous poplar transcript pairs (i.e., previously published poplar sequences with high sequence similarity to FLcDNAs reported in this study), 228 (79.2%) agreed well with regard to their ORF lengths and position of their start and stop codons (± ten amino acids; Figure 4). For the remaining pairs, the predicted 5' and/or 3' ORF ends did not match suggesting alternative start or stop codons, splice variants, or the possibility that one of the pair members was either truncated or had an incorrectly predicted ORF. When comparing the poplar FLcDNA collection to reciprocal matches from TAIR Arabidopsis peptides, we observed a similar number of 2,151 (77.5%) pairs with similar ORF lengths and positions of their starting methionine and stop codons (± ten amino acids; Figure 4). These results indicate the majority of the 4,664 poplar FLcDNAs represent true full-length transcripts with complete ORFs and correctly annotated start and stop codons.

### Mapping FLcDNAs to the poplar genome sequence to reassess gene prediction and to identify possible gaps in the genome assembly

**Figure 5:**
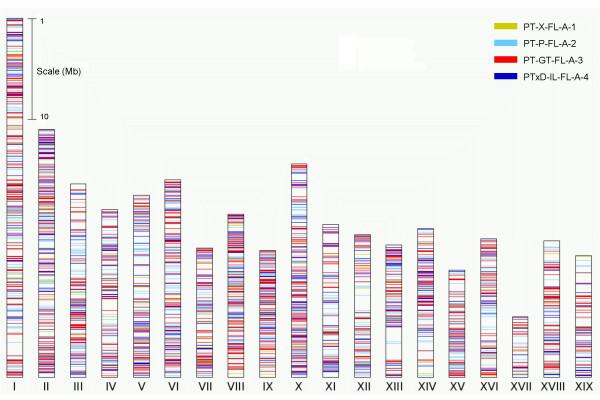
**Mapping FLcDNAs to the poplar genome**. 4,664 poplar FLcDNAs were aligned to the genome using BLAT with default parameters (match length ≥ 11 bp, BLAT score ≥ 30, sequence identity ≥ 90%). Prior to alignment, the 5' second strand primer adaptor sequences (SSPA) and polyA tails were removed. Among 4,642 poplar FLcDNAs that exceeded the minimal criteria for a match to the genome, 3,847 mapped to chromosomes whereas the remainder mapped to scaffold segments. Colored bars indicate the cDNA library of origin for those FLcDNAs mapping to one of the 19 poplar chromosomes. Applying a higher stringency threshold (sequence identity ≥ 95%, alignment coverage ≥ 95%), 4,487 or 96.2% of poplar FLcDNAs could be mapped to the genome.

As part of the poplar genome sequencing project [2], the poplar FLcDNAs were used to train a series of gene prediction algorithms to identify coding regions in the genome sequence. To reassess the effectiveness of gene prediction in the current genome assembly and to search for possible genome sequence gaps, we took two approaches: 1) BLAT [41] was utilized to map FLcDNAs to the assembled genome sequence, and 2) BLASTN was applied to align FLcDNAs with the 45,555 protein-coding gene loci predicted from the poplar genome sequence. Using BLAT, we mapped 4,642 poplar FLcDNAs (99.5%) to the genome at a minimum threshold (tile match length ≥ 11 bp, score ≥ 30, sequence identity ≥ 90%; Figure 5). From this set, 3,847 (82.9%) mapped to the 19 linkage groups (i.e., chromosomes) whereas the remainder mapped to scaffold segments that were not incorporated into the poplar genome sequence assembly. Examination of the linkage group location of FLcDNAs suggests a pattern of random distribution when grouped by cDNA library/tissue of origin, with an approximately even distribution of FLcDNAs throughout the genome (Figure 5). When we applied a more stringent similarity threshold (sequence identity ≥ 95%, alignment coverage ≥ 95%), the number of poplar FLcDNAs matching to the genome was only slightly reduced to 4,487 (96.2%).

In addition to BLAT analysis, we also compared the FLcDNAs with the 45,555 predicted protein-coding gene loci identified in the genome sequence using BLASTN and observed 4,452 (95.5%) matched at an E value < 1e^-50 ^(see Additional file 1). In order to identify possible sequence gaps in the 7.5× coverage genome, we searched for FLcDNAs lacking a stringent BLAT to the genome match and a BLASTN match (E value ≥ 1e^-50^) to the predicted gene models. This approach identified only 39 candidates, of which 20 (0.4%) FLcDNAs also had a strong match by BLASTN (E value < 1e^-50^) to one or more poplar ESTs in the public domain, excluding ESTs reported in this study (Table 2 and see Additional file 1), suggesting that these FLcDNAs represent expressed poplar genes that likely map to gap regions within the current genome draft. We cannot exclude the possibility that the remaining 19 FLcDNAs represent sequences from bacterial, fungal or insect species present on poplar tissues harvested for cDNA library construction, which were not filtered as contaminant sequences in our EST and FLcDNA processing procedures.

**Table 2:** Expressed FLcDNAs that identify possible gaps in the genome sequence assembly

Clone ID	GenBank ID	FLcDNA length (bp)	FL status/ORF size (aa)	NR BLASTP best match		dbEST BLASTN best match	
				GenBank accession, gene name, species	BLAST Score	GenBank accession, species	BLAST Score
WS0138_J20	EF148816	1444	FL/340	AAB39877.1, NMT1 protein, *Uromyces fabae*	1572	DN493922.1, *Populus tremula*	770
WS01313_D10	EF148323	1439	FL/363	At3g20790, oxidoreductase, *Arabidopsis thaliana*	1233	DN501083, *P. trichocarpa*	1318
WS0127_P01	EF148143	1237	FL/299	AAD01907, methenyltetrahydrofolate dehydrogenase, *Pisum sativum*	1213	CV131075.1, *P. deltoides*	1511
WS01231_K20	EF147482	1207	FL/256	At5g20060, phospholipase/carboxylesterase family, *A. thaliana*	1026	DV464443.2, *P. fremontii *× *P. angustifolia*	1479
WS0135_G15	EF148633	992	n.a.	No matches	n.a.	BU891205, *P. tremula*	240
WS01312_F21	EF148269	946	n.a.	No matches	n.a.	BI122644.1, *P. tremula *× *P. tremuloides*	729
WS01315_I11	EF148467	836	n.a.	No matches	n.a.	BU824948.1, *P. tremula *× *P. tremuloides*	339
WS01312_H02	EF148274	835	n.a.	No matches	n.a.	BU791223.1, *P. trichocarpa *× *P. deltoides*	779
WS01212_B01	EF146690	821	FL/88	BAB68268.1, drought-inducible protein, *Saccharum officinarum*	147	BU879805.1, *P. trichocarpa*	595
WS0122_E05	EF147284	739	FL/131	CAB80775.1, proline-rich protein, *A. thaliana*	340	BU866461.1, *P. tremula*	890
WS0122_O15	EF147357	736	FL/162	At4g10300, hypothetical protein, *A. thaliana*	444	CX181869.1, *Populus *× *canadensis*	1215
WS0113_C11	EF145750	722	FL/136	At3g12260, complex 1/LVR family protein, *A. thaliana*	426	BU879375.1, *P. trichocarpa*	1223
WS0125_P18	EF147919	596	3' trunc./70	AAF71823.1, pumilio domain protein, *P. tremula *× *P. tremuloides*	167	CX187487.1, *Populus *× *canadensis*	722
WS01123_K15	EF145357	483	n.a.	No matches	n.a.	CK319617.1, *P. deltoides*	268
WS01231_G04	EF147458	416	5' trunc./62	At3g18790, hypothetical protein, *A. thaliana*	200	CX184264.1, *Populus *× *canadensis*	543
WS0124_L22	EF147751	360	n.a.	No matches	n.a.	BI128250.1, *P. tremula *× *P. tremuloides*	494
WS0126_O09	EF148027	342	n.a.	No matches	n.a.	CF228572.1, *P. tremula *× *P. alba*	410
WS01118_P04	EF144846	300	n.a.	No matches	n.a.	CX184524.1, *Populus *× *canadensis*	242
WS0136_N09	EF148717	278	n.a.	No matches	n.a.	CX179364.1, *Populus *× *canadensis*	458
WS0138_I14	EF148811	231	n.a.	No matches	n.a.	CX170421.1, *P. deltoides*	228

To identify expressed genes that were not predicted in the original genome annotation [2], we searched among the set of 4,487 FLcDNAs with a stringent BLAT match to the genome that did not match to any of the 45,555 predicted gene models (E value ≥ 1e^-50^). This analysis revealed 173 FLcDNAs, 79 of which also showed strong similarity (E value < 1e^-50^) to one or more poplar ESTs in the public domain (see Additional file 1), suggesting that these 79 FLcDNAs represent expressed genes and possibly non-coding RNAs, that were missed by gene prediction software during the annotation of the poplar genome. The fact that these poplar transcripts had been missed could be due in part to the relatively short lengths of these 79 FLcDNAs (average FLcDNA and predicted ORF length of 555 bp and 67 bp, respectively; see Additional file 1).

### Comparative sequence annotation of poplar FLcDNAs against Arabidopsis and other plants identifies proteins unique to poplar

**Figure 6:**
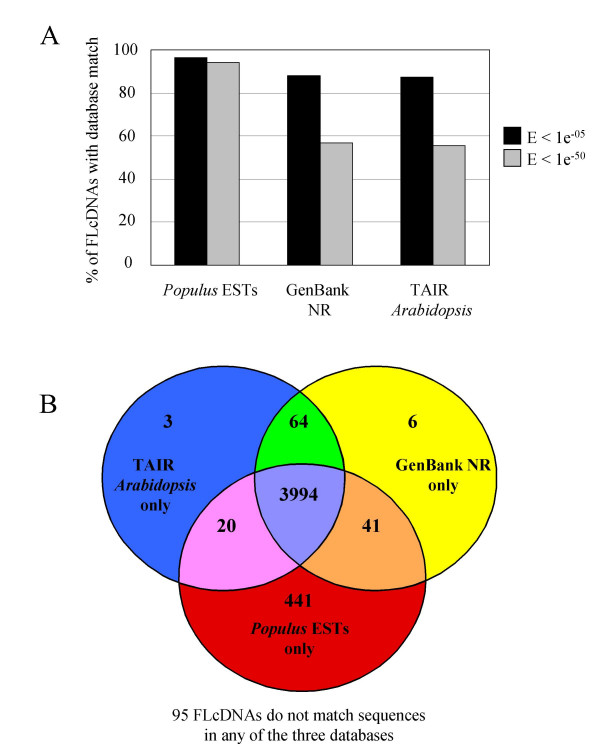
**Sequence annotation of 4,664 high-quality poplar FLcDNAs against published databases**. Panel A shows the percentage of FLcDNAs with similarity to entries in three databases using expect (E) value thresholds of < 1e^-05 ^and < 1e^-50^: matches to previously published poplar ESTs (i.e., ESTs available in GenBank, excluding ESTs from this study) identified by BLASTN; amino acid sequences in the non-redundant (NR) division of GenBank identified by BLASTX; and The Arabidopsis Information Resource (TAIR) non-redundant Arabidopsis peptide matches identified by BLASTX. Panel B shows a Venn diagram of distinct and overlapping patterns of sequence similarity against the three databases (public poplar ESTs, TAIR, NR) at a BLAST E value threshold of < 1e^-05^. At this threshold, 95 poplar FLcDNAs had no similarity to sequences in any of the databases examined.

Despite the growing research interest in poplar as a model angiosperm tree species and the recent completion of the poplar genome sequence, poplar still represents a difficult experimental system with relatively few functionally characterized proteins, compared to other established model systems such as Arabidopsis. Therefore, our effort of *in silico *annotation of poplar FLcDNAs was largely based on comparison with Arabidopsis together with the NR database of GenBank containing sequences from all plants, among other species. Using BLASTX, we found that the proportion of FLcDNAs with similarity to TAIR Arabidopsis proteins was 87.5% (4,081) at E value < 1e^-05 ^and 55.5% (2,590) at E value < 1e^-50 ^(Figure 6A). Similar values were obtained when using BLASTX to compare against peptides from other species in the NR division of GenBank (88.0% matches at E value < 1e^-05 ^and 56.9% matches at E value < 1e^-50^) (Figure 6A). As expected, the proportion of poplar FLcDNAs with sequence similarity to previously published poplar ESTs (i.e., ESTs available in the dbEST division of GenBank, excluding ESTs from this study) by BLASTN was very high, with 96.3% (4,496) and 94.3% (4,401) of FLcDNAs having matches with E values < 1e^-05 ^and < 1e^-50^, respectively (Figure 6A).

To identify genes that are potentially unique to poplar, we next examined the relationship of sequence similarity among the poplar FLcDNAs and best matching sequences in the TAIR Arabidopsis proteins, other NR database proteins (which includes all plant species), and previously published poplar EST datasets. Of the 4,664 poplar FLcDNAs, 3,994 (85.6%) had at least low sequence similarity to sequences in all three databases (E values < 1e^-05^; Figure 6B). Only 95 FLcDNAs had no similarity (E values ≥ 1e^-05^) to sequences in any of these databases; however, 87 of these strongly matched to the poplar genome using BLAT (sequence identity ≥ 95%, alignment coverage ≥ 95%). Our results suggest that these 87 genes that are represented with FLcDNAs and with poplar genomic sequences are new genes that have not previously been identified in other poplar EST collections or among genes in Arabidopsis and other plant species (see Additional file 1).

In addition, we also identified 536 poplar FLcDNAs (including the 95 FLcDNAs with no similarity to sequences in the three databases examined) with no similarity to Arabidopsis or NR proteins (E values ≥ 1e^-05^), of which 346 FLcDNAs matched with high similarity to both the poplar genome by BLAT and to previously published poplar ESTs by BLASTN (E values < 1e^-50^; Figure 6B and see Additional file 1). These poplar FLcDNAs could represent genes that were gained and then rapidly diverged in sequence since the recent whole genome duplication in poplar, or they may also represent non-coding RNAs or small peptides in poplar that share limited sequence similarity with other plants. The fact that these putative poplar-specific FLcDNAs do not share similarity with existing plant sequence data may also reflect the limited availability of sequence data from Salicaceae species closely related to poplar in the current NR database. To test these putatively poplar-specific FLcDNAs for known functional domains, we performed a search of the Pfam database [42]. At a threshold of E values < 1e^-05^, we identified 2,908 (62.3%) poplar FLcDNAs with similarity to a Pfam domain; however, among the collection of 346 putatively poplar-specific genes only 8 FLcDNAs in this set matched a Pfam domain (see Additional file 1). Domain matches included PF05162.3/ribosomal protein L41 (WS0112_A21, WS0116_F12, WS0124_J06, WS01230_B01, and W01118_I11), PF05160.3/DSS1/SEM1 family (WS0123_P21), PF06376.2/unknown function (WS0112_B13), and PF04689.3/DNA binding protein S1FA (WS01110_K04).

### Annotation of poplar FLcDNA transcripts affected by FTC herbivory

A major emphasis of the program that motivated the development and analysis of poplar FLcDNAs is the discovery of genes affected by insect attack. To identify herbivore-responsive genes among the poplar FLcDNAs, we first mapped the FLcDNA set onto a poplar 15.5 K microarray based on BLASTN comparison to ESTs spotted on the array. This microarray platform was previously used for profiling of the poplar leaf transcriptome affected by FTC larvae feeding [11]. Using a stringent similarity threshold of ≥ 95% identity over ≥ 95% alignment coverage, we identified 3,854 FLcDNAs that matched with 3,974 EST elements on the array (see Additional file 2). Although we did observe some cases of individual FLcDNAs mapping to multiple array elements, as well as multiple FLcDNAs mapping to the same array element, it should be noted that the *in silico *match stringency applied here is likely higher than the capability of cDNA microarrays to discriminate among highly similar transcripts by actual DNA hybridization. Next, we identified poplar FLcDNAs with a role in the response to insect attack by screening the 3,854 FLcDNAs against existing transcriptome data of differentially expressed (DE) genes in leaves that were exposed for 24 hours to FTC feeding [11]. This approach resulted in the identification of 129 and 24 FLcDNAs that were induced or repressed, respectively, in FTC-treated leaves compared to untreated control leaves (Tables 3 and 4) using the DE criteria of fold-change ≥ 2.0-fold, *P *value < 0.05 and *Q *value < 0.05. A complete list of expression data is provided [see Additional file 2]. Each of the 153 FLcDNAs was translated and evaluated for the presence of ORFs, and annotation was assigned based on manual examination of the highest scoring and most informative BLASTX matches in NR.

**Table 3:** FLcDNAs corresponding to transcripts most strongly induced by forest tent caterpillar (FTC) feeding [fold-change (FC) ≥ 2.0, *P *value < 0.05, *Q *value < 0.05]

				NR BLASTP best match		FTC feeding @ 24 h		
15.5 K Array ID	Matching FLcDNA ID	GenBank ID	FL status/ORF size (aa)	GenBank accession, gene name, species	BLAST score	FC	*P*	*Q*
WS0151_M13	WS0131_K04^a^	EF148503	FL/202	BAB85998.1, Kunitz trypsin inhibitor, *Populus nigra*	396	60.4	<0.001	<0.001
WS0132_F23	WS0133_O14^a^	EF148554	FL/202	BAB85997.1, Kunitz trypsin inhibitor, *P. nigra*	380	50.2	<0.001	<0.001
WS0134_B13	WS0134_B13	EF148557	FL/212	AAQ84217.1, Kunitz trypsin inhibitor, *Populus trichocarpa *× *deltoides*	387	46.2	<0.001	<0.001
WS0133_N23	WS0133_N23	EF148553	FL/197	CAJ21341.1, Kunitz trypsin inhibitor, *P. nigra*	383	38.8	<0.001	<0.001
WS0124_G12	WS0124_G12	EF147703	FL/159	AAQ08196.1, translation initiation factor 5A, *Hevea brasiliensis*	316	29.0	<0.001	<0.001
WS01223_D01	WS01223_D01	EF146918	FL/359	At1g74320, choline kinase, *Arabidopsis thaliana*	537	28.4	<0.001	<0.001
WS0134_E16	WS0134_E16	EF148571	5' trunc./124	AAA16342.1, vegetative storage protein, *P. trichocarpa *× *deltoides*	239	27.4	<0.001	<0.001
WS01120_O24	WS01120_O24	EF145143	3' trunc./56	At4g07960, putative glucosyltransferase, *A. thaliana*	72	26.4	<0.001	<0.001
WS01211_H19	WS01211_H19	EF146657	FL/337	CAN72815, hypothetical protein, *Vitis vinifera*	253	26.0	<0.001	<0.001
WS0121_J16	WS0122_N13	EF147347	FL/339	AAK01124.1, vegetative storage protein, *P. trichocarpa *× *deltoides*	509	25.4	<0.001	<0.001
WS0141_P05	WS0132_K10^a^	EF148516	FL/202	AAQ84216.1, Kunitz trypsin inhibitor, *Populus trichocarpa *× *deltoides*	386	22.7	<0.001	<0.001
WS01118_D16	WS01118_D16	EF144781	n.a.	No protein matches	n.a.	16.8	<0.001	<0.001
WS0168_C17	WS01119_J20	EF144899	FL/285	AAY43790.1, hypothetical protein, *Gossypium hirsutum*	77	16.0	<0.001	<0.001
WS01119_E18	WS01119_E18	EF144877	3' trunc./67	At5g61770, brix domain-containing protein, *A. thaliana*	85	15.7	<0.001	<0.001
WS0133_B24	WS0133_K20^a^	EF148543	FL/202	CAH59150.1, Kunitz trypsin inhibitor, *Populus tremula*	351	15.5	<0.001	<0.001
WS0155_D02	WS0138_H02^a^	EF148810	FL/251	BAB21610.2, mangrin/allene oxide cyclase, *Bruguiera sexangula*	336	14.4	<0.001	<0.001
WS0152_M24	WS0128_J15	EF148194	FL/91	At5g24165, hypothetical protein, *A. thaliana*	72	13.7	<0.001	<0.001
WS01118_N14	WS01118_N14	EF144837	frameshift/47	At4g27960, ubiquitin conjugating enzyme 9, *A. thaliana*	96	13.2	<0.001	<0.001
WS01212_M19	WS0128_D22	EF148166	FL/509	ABA01477.1, cytochrome P450, *Gossypium hirsutum*	726	12.3	<0.001	0.002
WS01211_N06	WS0118_O23^a^	EF146529	FL/225	ABS12347.1, dehydrin, *P. nigra*	167	11.8	<0.001	<0.001
WS0132_A15	WS01313_N19	EF148368	FL/396	At4g18550, lipase class 3 family protein, *A. thaliana*	385	11.6	<0.001	0.001
WS01212_B20	WS0128_L03	EF148205	FL/318	CAA73220.1, isoflavone reductase, *Citrus *× *paradise*	469	10.4	<0.001	<0.001
WS0122_C03	WS0122_C03	EF147271	FL/133	CAN82925.1, hypothetical protein, *V. vinifera*	114	9.2	<0.001	0.001
WS0113_H20	WS0113_H20	EF145803	n.a.	No protein matches	n.a.	8.8	<0.001	<0.001
WS0134_J14	WS0134_J14^a^	EF148597	FL/202	AAQ84216.1, Kunitz trypsin inhibitor, *P. trichocarpa *× *deltoides*	380	7.9	<0.001	<0.001
WS01120_N21	WS01120_N21	EF145138	n.a.	No protein matches	n.a.	6.9	<0.001	<0.001
WS0114_H12	WS0114_H12	EF145947	FL/252	At4g01470, major intrinsic family protein, *A. thaliana*	364	6.3	<0.001	<0.001
WS0126_E15	WS0126_E15	EF147963	FL/325	At1g30910, molybdenum cofactor sulfurase family protein, *A. thaliana*	444	6.2	<0.001	<0.001
WS0168_F14	WS01123_O20	EF145380	FL/217	At3g18030, phosphopantothenoyl cysteine decarboxylase, *A. thaliana*	350	6.2	<0.001	<0.001
PX0019_C05	PX0019_C05	EF144379	FL/214	AAF64453.1, heat-shock protein 90, *Euphorbia esula*	330	5.7	<0.001	<0.001
WS0205_K16	WS01214_G11	EF146815	FL/387	CAN71454.1, hypothetical protein, *V. vinifera*	682	5.6	<0.001	<0.001
WS0152_N17	WS0114_F10^a^	EF145928	FL/70	BAA03527.1, ATP synthase epsilon subunit, *Ipomoea batatas*	120	5.6	<0.001	0.001
WS01118_A11	WS0113_M04	EF145848	FL/97	At1g77710, ubiquitin-fold modifier precursor, *A. thaliana*	150	5.5	<0.001	<0.001
WS0132_L23	WS0132_L23	EF148518	FL/372	AAP87281.1, beta-1,3-glucanase, *Hevea brasiliensis*	540	5.4	<0.001	0.002
WS0124_C22	WS0124_C22	EF147658	5' trunc./142	CAA42660.1, luminal binding protein, *Nicotiana tabacum*	213	5.4	<0.001	<0.001
WS01116_C06	WS01123_N20	EF145376	FL/250	At4g38210, expansin A20 precursor, *A. thaliana*	351	5.2	<0.001	<0.001
WS0114_D04	WS01211_M02^a^	EF146676	FL/414	AAB71419.1, calreticulin, *Ricinus communis*	556	5.0	<0.001	<0.001
WS01117_O15	WS01117_O15	EF144759	FL/230	At4g11150, Vacuolar ATP synthase subunit E1, *A. thaliana*	295	4.7	<0.001	<0.001
WS0133_J24	WS0133_J24	EF148541	FL/177	At1g01250, AP2 transcription factor, *A. thaliana*	303	4.6	0.001	0.004
WS0148_P02	WS0127_F13	EF148073	5' trunc./283	At1g64660, methionine gamma-lyase, *A. thaliana*	424	4.5	<0.001	0.001
WS02010_D02	WS0126_C10^a^	EF147943	FL/68	NP_001066879.1, hypothetical protein, *Oryza sativa*	175	4.4	<0.001	<0.001
WS0155_H06	WS0125_E23	EF147828	FL/215	CAN69111.1, glutathione-S-transferase, *V. vinifera*	415	4.3	<0.001	<0.001
WS01119_L18	WS01119_L18	EF144906	FL/56	NP_001068325.1, 40S ribosomal protein, *O. sativa*	182	4.3	<0.001	<0.001
WS0134_F23	WS0134_F23	EF148579	FL/312	CAN79077.1, annexin, *V. vinifera*	575	4.2	<0.001	<0.001
WS0117_C05	WS0124_M24	EF147756	FL/538	AAA80588.1, calnexin, *Glycine max*	1231	4.1	<0.001	<0.001
WS0175_A23	WS01125_H02^a^	EF145504	FL/181	AAT08648.1, ADP-ribosylation factor, *Hyacinthus orientalis*	587	4.0	0.004	0.014
WS0153_O15	WS0135_A12	EF148616	FL/388	At4g24220, vein patterning 1, *A. thaliana*	711	4.0	<0.001	<0.001
WS0141_G12	WS01312_A02	EF148234	FL/273	At1g19180, hypothetical protein, *A. thaliana*	160	4.0	<0.001	0.003
WS0168_D23	WS01230_E07	EF147385	FL/420	ABD32854.1, hypothetical protein, *Medicago truncatula*	670	4.0	<0.001	0.001
WS0154_B02	WS01228_N21	EF147184	5' trunc./186	At5g07340, calnexin, *A. thaliana*	251	3.9	<0.001	<0.001
WS01116_D23	WS01116_D23	EF144634	FL/84	At3g60540, sec61beta family protein, *A. thaliana*	92	3.8	<0.001	<0.001
WS0117_O22	WS0117_O22^a^	EF146403	FL/68	At1g27330, hypothetical protein, *A. thaliana*	103	3.5	<0.001	<0.001
WS0122_A01	WS01227_N20	EF147117	FL/399	At1g74210, glycerophosphodiester phosphodiesterase, *A. thaliana*	606	3.5	<0.001	<0.001
WS0144_K08	WS01119_H21	EF144889	FL/358	ABQ10199.1, cysteine protease, *Actinidia deliciosa*	594	3.5	<0.001	<0.001
WS0147_I02	WS0125_D08	EF147814	FL/444	AAS79603.1, prephenate dehydratase, *Ipomoea trifida*	653	3.3	<0.001	0.001
WS0111_C18	WS0125_B22^a^	EF147800	FL/395	P47916, S-adenosyl methionine synthetase, *P. deltoides*	785	3.3	<0.001	0.001
WS0151_N14	WS0127_M05	EF148121	FL/485	Q01781, S-adenosylhomocysteine hydrolase, *Petroselinum crispum*	939	3.3	<0.001	<0.001
WS01212_P09	WS01212_P09	EF146734	FL/161	ABC47922.1, pathogenesis-related protein 1, *Malus *× *domestica*	236	3.2	0.005	0.016
PX0015_M10	PX0015_M10	EF144335	n.a.	No protein matches	n.a.	3.2	<0.001	<0.001
WS0111_A20	WS0111_A20	EF144935	FL/360	CAN67616.1, cupin family protein, *V. vinifera*	474	3.2	<0.001	<0.001
WS0117_P18	WS0117_P18	EF146411	FL/93	NP_001047293.1, hypoxia-responsive family protein, *O. sativa*	122	3.2	<0.001	<0.001
WS0131_J08	WS0131_J08	EF148502	FL/452	AAA70334.1, omega-3 fatty acid desaturase, *Sesamum indicum*	708	3.1	<0.001	<0.001
WS0173_J22	WS01229_P15	EF147254	frameshift/441	CAH05011.1, alpha-dioxygenase, *Pisum sativum*	679	3.1	<0.001	0.002
WS0151_H21	WS01314_F07^a^	EF148393	FL/505	AAB05641.1, protein disulphide isomerase, *R. communis*	786	3.1	<0.001	<0.001
WS0141_E06	WS0128_M17	EF148216	FL/338	CAN79663.1, hypothetical protein, *V. vinifera*	284	3.0	<0.001	<0.001
WS01211_D15	WS01211_D15	EF146643	FL/258	NP_001061550.1, 60S ribosomal protein L7A, *O. sativa*	398	3.0	0.004	0.012
WS01110_A05	WS01110_A05	EF144530	5' trunc./46	AAT45244.1, EPSP synthase, *Conyza canadensis*	87	3.0	<0.001	<0.001
WS0122_A21	WS0122_A21	EF147261	FL/349	At3g62600, DNAJ heat shock family protein, *A. thaliana*	542	3.0	<0.001	<0.001
WS0154_D16	PX0019_K19	EF144475	FL/172	ABL67655.1, cyclophilin, *Citrus cv. Shiranuhi*	303	3.0	<0.001	<0.001
WS0114_N12	WS0114_N12	EF146003	5' trunc./243	AAU08208.1, chloroplast ferritin precursor, *Vigna angularis*	357	3.0	0.001	0.007
WS0153_O16	WS0136_K07^a^	EF148708	FL/113	CAA40072.1, hypothetical protein, *P. trichocarpa *× *deltoides*	225	2.9	<0.001	<0.001
WS01117_D04	WS01117_D04	EF144703	FL/137	CAN73155.1, hypothetical protein, *V. vinifera*	110	2.9	<0.001	<0.001
WS01120_A02	WS01120_A02	EF145080	5' trunc./105	At1g03010, phototropic-responsive NPH3 family protein, *A. thaliana*	177	2.8	<0.001	0.001
WS0178_L06	WS01211_M01	EF146675	FL/415	NP_001064428.1, no apical meristem transcription factor, *O. sativa*	98	2.8	<0.001	0.001
WS0143_C23	WS01228_M23^a^	EF147179	FL/212	ABB89210.1, dehydroascorbate reductase, *S. indicum*	343	2.7	<0.001	<0.001
WS0127_I09	WS0127_I09	EF148095	FL/235	CAB77025.1, Rho GDP dissociation inhibitor, *N. tabacum*	294	2.7	0.003	0.012
PX0015_K10	PX0015_K10	EF144326	3' trunc./65	At2g15590, hypothetical protein, *A. thaliana*	39	2.7	0.001	0.004
WS0152_M05	WS01111_A23	EF144570	FL/125	At1g69230, nitrilase-associated protein, *A. thaliana*	80	2.7	0.001	0.006
WS0134_H19	WS0134_H19	EF148589	FL/461	At5g28237. tryptophan synthase, *A. thaliana*	579	2.7	<0.001	0.001
WS0122_P22	WS0122_P22	EF147367	5' trunc./46	AAS89832.1, flavonoid 3-O-glucosyltransferase, *Fragaria *× *ananassa*	47	2.6	0.009	0.023
WS0113_E03	WS0113_E03	EF145764	5' trunc./130	At1g73600, phosphoethanolamine N-methyltransferase, *A. thaliana*	198	2.6	<0.001	0.001
WS02012_L20	WS01212_L02^a^	EF146720	FL/440	AAV50009.1, N-hydroxycinnamoyl/benzoyltransferase, *Malus *× *domestica*	451	2.5	<0.001	0.001
WS0116_I22	WS01119_O01^a^	EF144919	FL/212	ABB89210.1, dehydroascorbate reductase, *S. indicum*	360	2.5	<0.001	0.001
WS0128_C01	WS0128_C01	EF148156	FL/205	CAC85245.1, salt tolerance protein, *Beta vulgaris*	246	2.5	0.001	0.005
PX0011_E19	PX0011_C19	EF144204	FL/341	At1g10840, eukaryotic translation initiation factor subunit 3, *A. thaliana*	573	2.5	<0.001	0.002
WS0128_M01	WS0128_M01	EF148209	5' trunc./197	ABN08481.1, homeodomain-related, *M. truncatula*	103	2.4	<0.001	0.003
WS01126_B13	WS01126_B13	EF145551	3' trunc./136	CAN77060.1, ubiquitin activating enzyme, *V. vinifera*	239	2.4	0.017	0.035
WS01125_E14	WS01125_E14^a^	EF145493	FL/207	NP_001058535.1, cyclophilin, *O. sativa*	340	2.4	<0.001	0.001
WS01218_P22	WS01120_G07^a^	EF145102	FL/170	NP_001050870.1, glycine-rich RNA-binding protein, *O. sativa*	144	2.4	0.004	0.013
WS01117_L06	WS01117_L06	EF144744	frameshift/136	NP_001046690.1, ribosomal protein L10A, *O. sativa*	171	2.4	<0.001	<0.001
WS01117_E15	WS01117_E15	EF144711	n.a.	No protein matches	n.a.	2.4	<0.001	0.001
WS01110_A14	WS0122_K19	EF147330	FL/476	AAF18411.1, integral membrane protein, *Phaseolus vulgaris*	897	2.4	<0.001	<0.001
WS0156_A21	WS0127_G12^a^	EF148080	n.a.	No protein matches	n.a.	2.4	0.017	0.035
WS0127_G19	WS0127_G19	EF148082	frameshift/251	At4g11640, serine racemase, *A. thaliana*	354	2.4	<0.001	0.002
WS0112_O04	WS0112_O04	EF145713	5' trunc./566	ABS01352.1, methionine synthase, *Carica papaya*	1073	2.4	<0.001	0.001
WS0155_E17	WS01212_I06^a^	EF146705	FL/363	ABM67589.1, flavanone 3-hydroxylase, *V. vinifera*	645	2.4	0.003	0.012
WS0168_M07	WS0137_H13^a^	EF148760	FL/62	ABF98145.1, hypothetical protein, *O. sativa*	57	2.4	<0.001	0.003
WS0119_H18	WS0117_P08	EF146405	5' trunc./188	CAN83141.1, hypothetical protein, *V. vinifera*	218	2.3	<0.001	0.003
WS0157_L22	WS0128_B17	EF148154	5' trunc./388	CAN76057.1, glucosyltransferase, *V. vinifera*	411	2.3	0.002	0.008
WS0185_E12	WS0124_A18	EF147646	FL/285	CAH60723.1, aquaporin, *P. tremula *× *tremuloides*	488	2.3	0.001	0.007
WS0125_I01	WS0125_I01	EF147858	FL/477	BAA36972.1, flavonoid 3-O-galactosyl transferase, *Vigna mungo*	442	2.3	0.003	0.011
PX0019_C07	PX0019_C07	EF144380	5' trunc./222	CAN74465.1, hypothetical protein, *V. vinifera*	369	2.3	0.015	0.033
WS01111_E24	WS0113_P06	EF145877	FL/290	AAN32641.1, short-chain alcohol dehydrogenase, *Solanum tuberosum*	399	2.3	<0.001	0.003
WS01212_B14	WS01214_D06^a^	EF146806	FL/363	ABM67589.1, flavanone 3-hydroxylase, *V. vinifera*	644	2.3	0.003	0.011
WS0181_A04	WS01312_M14	EF148294	frameshift/232	CAN74806, bZIP transcription factor, *V. vinifera*	152	2.3	0.002	0.009
WS0116_F22	WS0116_F22	EF146228	frameshift/239	At3g05290, mitochondrial substrate carrier protein, *A. thaliana*	283	2.3	0.004	0.013
WS01121_C12	WS01121_C12	EF145159	FL/216	At2g25110, MIR domain-containing protein, *A. thaliana*	349	2.3	<0.001	<0.001
WS01214_P11	WS01214_P11	EF146849	FL/219	ABL84692, glutathione S-transferase, *V. vinifera*	345	2.3	0.002	0.009
WS0128_G16	WS01228_N10	EF147182	FL/207	AAN03471.1, hypothetical protein, *G. max*	99	2.2	<0.001	<0.001
WS0209_J01	WS0135_O22	EF148667	FL/318	AAG23965.1, endochitinase, *Vigna sesquipedalis*	461	2.2	0.001	0.004
WS01119_M12	WS01110_H18	EF144553	FL/118	At5g04750, F1F0-ATPase inhibitor protein, *A. thaliana*	52	2.2	<0.001	<0.001
WS0205_L05	WS01228_D08	EF147142	frameshift/233	AAX85981.1, NAC4 protein, *G. max*	362	2.2	0.019	0.038
WS0123_D13	WS0137_E08	EF148737	FL/533	At5g58270, STARK1 ATPase, half ABC transporter, *A. thaliana*	642	2.2	<0.001	<0.001
WS0112_P02	WS0116_L21	EF146273	FL/145	At5g27670, histone 2A, *A. thaliana*	196	2.2	<0.001	0.002
WS01214_A14	WS01225_E15	EF146945	FL/330	At5g07010, sulfotransferase family protein, *A. thaliana*	394	2.2	0.002	0.009
WS01211_G15	WS01211_G15	EF146653	FL/507	AAL24049.1, cytochrome P450, *Citrus sinensis*	677	2.2	<0.001	0.002
WS0123_E09	WS0123_E09	EF147535	FL/210	ABB89210.1, dehydroascorbate reductase, *S. indicum*	332	2.2	<0.001	<0.001
WS0114_N11	WS0114_N11	EF146002	5' trunc./313	AAF73006.1, NADP-dependent malic enzyme, *R. communis*	450	2.1	<0.001	<0.001
WS0154_G22	WS0122_L10	EF147335	5' trunc./381	CAN74204.1, hypothetical protein, *V. vinifera*	535	2.1	0.001	0.005
WS0181_N15	WS0133_H05	EF148536	FL/283	ABG73415.1, chloroplast pigment-binding protein, *N. tabacum*	496	2.1	<0.001	0.001
WS0131_L08	WS0137_P12^a^	EF148792	FL/214	NP_001060368.1, emp24/gp25L/p24 transmembrane protein, *O. sativa*	288	2.1	<0.001	<0.001
WS0124_N24	WS0124_N24	EF147765	FL/584	NP_001048852.1, acyl-activating enzyme 11, *O. sativa*	750	2.1	0.017	0.036
WS0116_E14	WS0116_E14	EF146213	n.a.	No protein matches	n.a.	2.1	0.001	0.004
WS0128_N06	WS0128_N06	EF148221	FL/257	At4g18260, cytochrome b-561, *A. thaliana*	294	2.1	0.005	0.016
WS01122_N10	WS01122_N10	EF145286	FL/91	At1g62440, leucine-rich repeat extensin, *A. thaliana*	107	2.0	0.010	0.025
WS01214_M13	WS01214_M13	EF146841	FL/378	At5g45670, GDSL-motif/hydrolase family protein, *A. thaliana*	298	2.0	<0.001	0.001
WS01213_H17	WS01213_H17	EF146756	FL/597	At4g34200, phosphoglycerate dehydrogenase, *A. thaliana*	884	2.0	<0.001	0.003
WS01122_N02	WS01231_J04^a^	EF147472	FL/196	XP_001334748.1, hypothetical protein, *Danio rerio*	59	2.0	0.003	0.010
WS0156_F12	WS0118_O10	EF146525	FL/102	At2g18400, ribosomal protein L6, *A. thaliana*	165	2.0	<0.001	<0.001

^a^Multiple FLcDNAs match to the same microarray EST, a complete list of matching FLcDNAs is provided elsewhere [see Additional file 2].

**Table 4:** FLcDNAs corresponding to transcripts most strongly repressed by forest tent caterpillar (FTC) feeding [fold-change (FC) ≥ 2.0, *P *value < 0.05, *Q *value < 0.05]

				NR BLASTP best match		FTC feeding @ 24 h		
15.5 K Array ID	Matching FLcDNA ID	GenBank ID	FL status/ORF size (aa)	GenBank accession, gene name, species	BLAST score	FC	*P*	*Q*
WS0162_B18	WS01227_D07	EF147075	FL/465	AAX84673.1, cysteine protease, *Manihot esculenta*	782	0.33	<0.001	<0.001
WS0112_D20	WS0112_D20	EF145637	FL/99	At1g67910, hypothetical protein, *Arabidopsis thaliana*	69	0.34	<0.001	0.001
WS0126_C06	WS0126_C06	EF147942	FL/121	At2g45180, protease inhibitor/lipid transfer protein, *A. thaliana*	108	0.34	0.018	0.038
WS0131_P03	WS0131_P03^a^	EF148510	FL/303	CAN63090.1, zinc finger transcription factor, *Vitis vinifera*	135	0.36	<0.001	0.001
WS0178_F11	WS01228_M08	EF147174	5' trunc./106	At1g22770, gigantea protein, *A. thaliana*	150	0.38	<0.001	0.002
WS0127_F15	WS0127_F15	EF148074	FL/173	CAN68427.1, hypothetical protein, *V. vinifera*	207	0.40	<0.001	0.001
WS0121_B24	WS0128_M21	EF148217	FL/139	AAU03358.1, acyl carrier protein, *Lycopersicon esculentum*	119	0.41	<0.001	<0.001
WS0147_J04	WS0134_M10	EF148605	n.a.	No protein matches	n.a.	0.41	0.004	0.014
WS0158_G10	WS0128_E13	EF148173	5' trunc./628	At1g56070, elongation factor, *A. thaliana*	1239	0.41	0.001	0.005
WS0152_E14	WS0112_O08^a^	EF145715	FL/252	ABH09330.1, aquaporin, *V. vinifera*	375	0.42	<0.001	0.003
WS0143_B24	WS01227_O15	EF147121	FL/267	At1g06460, small heat shock protein, *A. thaliana*	146	0.42	<0.001	0.001
WS0127_G18	WS0127_G18	EF148081	n.a.	No protein matches	n.a.	0.43	<0.001	<0.001
WS0182_D02	WS01226_N23	EF147055	FL/335	CAN75691.1, methyltransferase, *V. vinifera*	534	0.43	0.001	0.005
WS0124_D16	WS0124_D16	EF147668	FL/164	At3g62550, universal stress protein, *A. thaliana*	188	0.44	<0.001	0.001
WS0163_G24	WS0115_E02	EF146059	FL/341	AAD56659.1, malate dehydrogenase, *Glycine max*	566	0.45	0.003	0.010
WS0175_O14	WS01313_J01^a^	EF148349	FL/239	CAN63226.1, hypothetical protein, *V. vinifera*	313	0.45	<0.001	0.001
WS0178_N22	WS01111_H24	EF144589	FL/161	ABG27020.1, SKP1-like ubiquitin-protein ligase, *Medicago truncatula*	219	0.46	<0.001	<0.001
WS0121_H19	WS0121_H19	EF146882	FL/350	AAW66657.1, thiamine biosynthetic enzyme, *Picrorhiza kurrooa*	539	0.48	0.005	0.016
WS0206_B21	WS0131_B11	EF148494	FL/133	CAA59409.1, photosystem II reaction center protein, *Spinacia oleracea*	140	0.48	0.001	0.006
WS0155_M12	WS0136_E20	EF148683	FL/234	CAN60736.1, hypothetical protein, *V. vinifera*	313	0.48	0.001	0.007
WS0152_F02	WS01117_K24	EF144742	FL/384	CAN83255.1, CCCH-type zinc finger protein, *V. vinifera*	432	0.49	<0.001	0.002
WS01224_P10	WS0124_L08^a^	EF147742	FL/137	CAA28450.1, photosystem II 10 kDa polypeptide, *Solanum tuberosum*	191	0.49	<0.001	0.003
WS0115_N05	WS0115_N05	EF146146	FL/250	AAM21317.1, auxin-regulated protein, *Populus tremula *× *tremuloides*	449	0.50	0.005	0.016
WS0125_F02	WS0125_F02	EF147829	FL/516	At1g60590, polygalacturonase, *A. thaliana*	715	0.50	0.001	0.005

^a^Multiple FLcDNAs match to the same microarray EST, a complete list of matching FLcDNAs is provided elsewhere [see Additional file 2].

Among FTC-induced transcripts represented with FLcDNAs, we identified a large number of defense-related and stress response proteins such as chitinases, Kunitz protease inhibitors, dehydrins, beta-1,3-glucanases, pathogenesis related protein PR-1, and glutathione-S-transferase (Table 3). Several classes of transcription factors (TFs) were also strongly affected by FTC feeding such as bZIP domain TFs, NAC domain TFs, NAM domain TFs and ethylene response factor TFs. A number of genes associated with signaling were also strongly affected by FTC feeding, including allene oxide cyclase involved in jasmonate formation and calreticulin associated with calcium signaling. We also observed a substantial number of FLcDNAs annotated as involved in phenolic metabolism, particularly flavonoid biosynthesis, including isoflavone reductase, EPSP synthase, flavonoid 3-O-glycosyl transferase and flavanone 3-hydroxylase, along with several cytochrome P450s of unknown function (Table 3). Among the FTC-repressed transcripts represented with FLcDNAs, we observed photosystem II proteins associated with photosynthesis, malate dehydrogenase and thiamine biosynthesis enzyme associated with primary metabolism, several zinc finger TFs, and stress-responsive proteins such as small heat shock and universal stress proteins (Table 4). Twenty two of the 153 FTC-responsive genes represented with FLcDNAs matched to hypothetical proteins of unknown function and nine have no obvious similarity to any proteins in the NR database.

## Discussion

Previous studies using the biotinylated CAP trapper method for FLcDNA library construction have demonstrated this technique to be highly effective for capturing predominantly true full-length clones in large-scale projects [24,25,27]. In this study, we generated a set of 4,664 FLcDNAs, which represents the third largest plant FLcDNA resource published to date, behind only Arabidopsis and rice. CAP3 clustering and assembly indicates that more than 85% of the FLcDNAs are non-redundant within this collection. The average sequence length, ORF and UTR sizes of the poplar FLcDNAs were comparable to those observed with the CAP trapper-derived FLcDNA collections for maize [27], Arabidopsis [40] and rice [24], and were also very similar to the *ab initio *predicted reference genes in the poplar genome sequence [2]. Applying a reciprocal BLAST strategy, we demonstrated that among FLcDNAs with high sequence similarity to known Arabidopsis peptides and/or previously published poplar FLcDNAs, nearly 80% had similar ORF lengths and starting methionine and stop codon positions. Collectively, these data show that the poplar FLcDNA libraries are of high quality and that our clone selection strategy combined with the CAP trapper method was effective in capturing *bona fide *FLcDNAs from poplar.

Comparison of poplar FLcDNAs and the poplar genome sequence assembly confirmed both the overall high accuracy of the current genome assembly, as well as the quality of the FLcDNA resource described here. However, as has been previously demonstrated with efforts to identify the complete catalogue of genes in Arabidopsis and rice, gene prediction and genome assembly is an iterative process. The results reported here for the mapping of FLcDNAs to the poplar genome sequence reveal opportunities for improvement of the genome sequence assembly (i.e., targeting apparent gaps for re-sequencing), as well as opportunities to further improve tools for the *in silico *prediction of genes. To address the discovery of apparent gaps in the genome assembly, the availability of 39 FLcDNAs that are not covered in the current assembly could be used to target BAC clones for re-sequencing and filling of gap regions. Similarly, the discovery of 173 FLcDNAs that do not have corresponding gene predictions in the current genome annotation may provide an opportunity to further improve gene prediction tools for poplar. Algorithms used for gene prediction in the poplar genome sequence assembly could be tested with these 173 FLcDNAs to find out why they may have initially been missed. If this leads to an improvement of prediction tools, the assembled genome sequence could be tested with the modified tools to identify additional genes.

The comparative sequence annotation of poplar FLcDNAs against Arabidopsis, the NR database, and previously published poplar ESTs revealed that *ca*. 88% of poplar FLcDNAs showed similarity to sequences in Arabidopsis or other plants. Many of the *ca*. 11.5% of poplar FLcDNAs without significant sequence similarity in Arabidopsis or other plants are supported with evidence of gene expression in the form of previously published poplar ESTs and matching the poplar genome sequence, thus excluding the possibility that they are artifacts of cDNA library construction. The discovery of poplar FLcDNAs without matches in other plant species is also in agreement with previous analysis of the poplar genome sequence where 11% of predicted proteins had no similarity to proteins in the NR database and 12% had no similarity to Arabidopsis proteins [2]. For comparison, only 64% of the 28,444 ORFs derived from rice FLcDNAs showed significant similarity to coding sequences predicted from the Arabidopsis genome and conversely, only 75% of Arabidopsis coding sequences had similarity to rice FLcDNAs [24]. These findings suggest that a substantial proportion of protein-coding sequences are not conserved among all plant species. The putative poplar-specific genes could be the product of past local or whole genome duplications in the lineage that led to extant poplar species [2,43] followed by sequence divergence [44,45]. Furthermore, *ca*. 2% of poplar FLcDNAs did not contain a predicted ORF suggesting these putative poplar-specific genes likely encode non-coding RNAs (i.e., rRNAs, tRNAs, snoRNAs etc.).

## Conclusion

We developed a large FLcDNA resource of high sequence quality and low-level redundancy that facilitated the discovery of a substantial number of genes not present among the published sequences of other plant species, and that also facilitated the discovery of several hundred insect-affected genes in the poplar leaf transcriptome that were represented by FLcDNAs. The newly established poplar FLcDNA resource will be valuable for further improvement of the poplar genome assembly, annotation of protein-coding regions, and for functional and comparative analysis of poplar genes. Specifically, the identification of FLcDNAs that are not covered in the current genome assembly or that were not predicted during the genome annotation provides opportunities to further refine the current genome assembly. The availability of a large collection of FLcDNAs that show altered gene expression following insect herbivory affords more rapid characterization of the role of these genes in poplar biotic interactions.

## Methods

### Full-length cDNA libraries

Plant materials used in the construction of cDNA libraries are described in Table 1. Isolation of total and poly(A)^+ ^RNA are described elsewhere (see Additional file 3). FLcDNA libraries were directionally constructed (5' *Sst*I and 3' *Xho*I) according to published methods [46,47], with modifications described in detail elsewhere (see Additional file 3).

### DNA sequencing and sequence filtering

Details of bacterial transformation with plasmids, clone handling, DNA purification and evaluation, and DNA sequencing are provided elsewhere (see Additional file 3). Sequences from each cDNA library were closely monitored to assess library complexity and sequence quality. DNA sequence chromatograms were processed using the PHRED software (versions 0.000925.c and 0.020425.c) [48,49]. Sequences were quality-trimmed according to the high-quality (hq) contiguous region determined by PHRED and vector-trimmed using CROSS_MATCH software [50]. Sequences with less than 100 quality bases (Phred 20 or better) after trimming and sequences having polyA tails of ≥ 100 bases were removed from analysis. Also removed were sequences representing bacterial, yeast or fungal contaminations identified by BLAST searches [51,52] against *E. coli *K12 DNA sequence (GI: 6626251), *Saccharomyces cerevisiae *[53], *Aspergillus nidulans *(TIGR ANGI.060302), and *Agrobacterium tumefaciens *(custom database generated using SRS, Lion Biosciences). Sequences were also compared to the GenBank NR database using BLASTX. Top ranked BLAST hits involving other non-plant species and with E values < 1e^-10 ^were classified as contaminants and removed prior to EST assembly.

### Selection of candidate FLcDNA clones and sequencing strategy

All 3'-end ESTs remaining after filtering were clustered and assembled using CAP3 [39] (assembly criteria: 95% identity, 40 bp window). The resulting contigs and singletons were defined as the PUT set. PUTs with a cDNA clone from a FLcDNA library were selected as candidates for complete insert sequencing (Figure 1). Candidate clones from FLcDNA libraries were single-pass sequenced from both 3'- and 5'-ends and both sequences were used for subsequent clone selection. Next, clones were screened for the presence of a polyA tail (3'-end EST) and the second-strand primer adaptor (SSPA; 5'-ACTAGTTTAATTAAATTAATCCCCCCCCCCC-3'; 5'-end EST). Clones lacking either of these features were eliminated. A polyA tail was defined as at least 12 consecutive, or 14 of 15 "A" residues within the last 30 nt of the 3'-end EST (5' to 3'). The presence of the SSPA was detected using the Needleman-Wunsch algorithm limiting the search to the first 30 nt of the 5'-end EST (5' to 3'). The SSPA was defined as eight consecutive "C" residues and a > 80% match to the remaining sequence (5'-ACTAGTTTAATTAAATTAAT-3'). In each case, the algorithms used to detect the 5' and 3' clone features were set to produce maximal sensitivity while maintaining a 0% false positive rate, as determined using test data sets. Candidate clones for which either of the initial 5'-end or 3'-end EST reads had a Phred20 quality length of < 100 nt were also excluded. Finally, candidate clones were compared to poplar ESTs in the public domain (excluding ESTs from this collection; BLASTN match E < 1e^-80^) to identify candidate FLcDNAs potentially truncated at the 5' end of the transcript relative to a matching EST. Any clone with a 5' end that was > 100 nt shorter than the matching public EST was excluded. For each PUT represented by multiple candidate clones after filtering, the clone with the longest 5' sequence was selected for complete insert sequencing. Insert sizing performed on 4,848 of 5,926 candidate clones using colony PCR with vector primers and standard gel electrophoresis revealed an average insert size of *ca*. 1,085 bp. Based on this information, a sequencing strategy emphasizing the use of end reads was chosen.

### Sequence finishing of FLcDNA clones

FLcDNA clones selected for complete sequence finishing were rearrayed into 384-well plates, followed by an additional round of 5'-end and 3'-end sequencing using vector primers. All end reads from an individual clone were then assembled using PHRAP (version 0990329) [48-50]. To meet our sequence quality criteria, the resulting clone consensus sequence was required to achieve a minimum average score of Phred35, with each base position having a minimum score of Phred30. Each base position also required at least two sequence reads, of minimum Phred20, that were in agreement with the consensus sequence (i.e., no high-quality discrepancies). Clones that did not meet these finishing criteria after two rounds of end read sequencing were then subjected to successive rounds of sequencing using custom primers designed using the Consed graphical tool version 14 [54] until the required quality levels were achieved. Regardless of the finishing strategy, all clones that did not meet the minimum finishing criteria according to an automated pipeline were flagged for manual examination. Clones were aborted if they were manually verified to lack the minimum finishing criteria after three rounds of custom primer design, were identified as chimeric sequences, or were refractory to sequence finishing due to the presence of a "hard-stop". FLcDNA sequences have been deposited in the NR division of GenBank [EF144175 to EF148838].

### Gene expression meta-analysis of FLcDNAs

Poplar FLcDNA sequences were mapped to a cDNA microarray containing 15,496 poplar ESTs [[11]; Gene Expression Omnibus (GEO) platform number GPL5921] using BLASTN with a stringent threshold of ≥ 95% identity over ≥ 95% of alignment coverage. To identify FLcDNAs that were DE following FTC feeding, FLcDNAs mapping to the microarray were matched to an existing microarray dataset that examined gene expression in hybrid poplar leaves 24 hours after continuous FTC feeding ([11]; GEO series number GSE9522).

## Authors' contributions

This study was conceived and directed by SGR, CJD and JB. Full-length cDNA libraries were developed by SGR, DC and NK. Data was analyzed by SGR, HJEC and RK with assistance from the coauthors. LG conducted DNA sequencing at the ORNL under the direction of GAT. RAH, SJMJ and MM directed sequencing and bioinformatics work at the GSC. SGR, HJEC and JB wrote the paper. All authors read and approved the final manuscript.

## Supplementary Material

Additional file 1 Full-length cDNA inventory. Predicted protein-coding features and annotation for the poplar full-length cDNA collection. Click here for file

Additional file 2 Microarray dataset. Poplar FLcDNAs mapped to the genome-wide transcript profile of poplar leaves 24 h after the onset of forest tent caterpillar feeding using a 15.5 K array. Click here for file

Additional file 3 Supplemental methods. Poplar methods for RNA isolation, full-length cDNA library construction, bacterial transformation with plasmids, clone handling, DNA purification and evaluation, and DNA sequencing are provided. Click here for file
